# Sensor-Based Extraction Approaches of In-Vehicle Information for Driver Behavior Analysis

**DOI:** 10.3390/s20185197

**Published:** 2020-09-11

**Authors:** Beomjun Kim, Yunju Baek

**Affiliations:** School of Computer Science and Engineering, Pusan National University, Busan 46241, Korea; beomjun.kim@eslab.re.kr

**Keywords:** in-vehicle sensor, controller area network, vehicular information, reverse engineering, vehicle state estimation

## Abstract

Advances in vehicle technology have resulted in the development of vehicles equipped with sensors to acquire standardized information such as engine speed and vehicle speed from the in-vehicle controller area network (CAN) system. However, there are challenges in acquiring proprietary information from CAN frames, such as the brake pedal and steering wheel operation, which are essential for driver behavior analysis. Such information extraction requires electronic control unit identifier analysis and accompanying data interpretation. In this paper, we present a system for the automatic extraction of proprietary in-vehicle information using sensor data correlated with the desired information. First, the proposed system estimates the vehicle’s driving status through threshold-, random forest-, and long short-term memory-based techniques using inertial measurement unit and global positioning system values. Then, the system segments in-vehicle CAN frames using the estimation and evaluates each segment with our scoring method to select suitable candidates by examining the similarity between each candidate and its estimation through the suggested distance matching technique. We conduct comprehensive experiments of the proposed system using real vehicles in an urban environment. Performance evaluation shows that the estimation accuracy of the driving condition is 84.20%, and the extraction accuracy of the in-vehicle information is 82.31%, which implies that the presented approaches are quite feasible for automatic extraction of proprietary in-vehicle information.

## 1. Introduction

Advancements in the automotive and semiconductor industries have led to the development of increasingly sophisticated vehicles. There has also been a recent interest in smart vehicle technologies such as advanced driver assistance systems (ADASs) and autonomous driving, and studies have been conducted on various related technologies [1,2]. These studies are concerned with recognizing or predicting human driving patterns, drowsy driving, driver behavior, and driving intentions [3,4,5,6,7,8]. There are two representative approaches to studies on driver technologies, namely use of a vehicle simulator and experimenting with a real vehicle [9]. Simulator-based studies [10,11] facilitate the acquisition of information difficult to obtain under real driving conditions, as well as data on dangerous driving conditions such as drowsy or drunk driving. The findings of the simulator research can be extended to the actual driving environment [12]. Simulator-based research is mainly used for research on driver behavior; however, the drivers might behave differently in a simulation environment which is considered safe and flexible.

In studies on smart vehicle technology using real vehicles, real-time information is collected via various types of sensors and devices such as an internal camera that captures the face and eyes of the driver [7] or an external camera that captures images of the road [5,13]. Light detection and ranging (LiDAR) and radar technologies may also be used to monitor the environment [14,15]. Some studies also utilize wearable sensors to monitor the driver’s condition [16]. As an alternative to installing additional sensors in vehicles, some researchers have utilized conventional smartphones as sensing devices [17,18,19,20,21]. The use of a smartphone as a sensor is inexpensive, but the collected information is limited to the functions supported by the smartphone. Typical sensors in a smartphone are acceleration sensors, gyro sensors, and global positioning systems (GPSs).

Vehicle components that can be used to acquire information without additional sensors include the On-Board Diagnostics II (OBD-II) connector, with pins for controller area network (CAN) communication. CAN is designed with international standards for communication between electronic control units (ECUs) in vehicles. The CAN network uses two lines, CAN_H and CAN_L, to form a bus structure, that enables the simultaneous connection of multiple ECUs, thereby reducing the vehicle’s wiring cost. The physical and data link layers were established as per ISO 11898 standards. The destination is set using a message-based protocol rather than a physical address. Each ECU has an identifier (ID) composed of 11 or 29 bits based on their priority. All CAN frames are prioritized based on the ID; thus, if two nodes transmit a message simultaneously, a message with a higher priority is transmitted first, and a message with a lower priority is delayed. The data field of the CAN frame consists of 0 to 8 bytes. Thus, a single CAN frame can hold up to 64 individual signals.

Another example of an in-vehicle network is the automotive ethernet-based communication. ISO 13400, a standard for diagnosing vehicle conditions through Diagnostic communication over Internet Protocol (DoIP), has been established. In addition, automotive ethernet using IEEE’s Time-Sensitive Networking (TSN) is a state-of-the-art option for driver assistance [22]. IETF’s Deterministic Networking (DetNet) has been extended to Layer 3, unlike TSN, that considers only OSI model Layer 2 [23]. ISO 13400-3 allocates pins 3, 8, 11, 12, and 13 of the SAE J1962 connector for ethernet. However, compared with the widely used CAN, the emerging automotive ethernet technology is limitedly adopted.

The On-Board Diagnostics (OBD) is a device that diagnoses the vehicle condition for quick failure identification. The OBD-II connector is a representative interface of the OBD standardized by SAE J1962. In its standard form, pins 6 and 14 are reserved for CAN communication. The SAE J1979 standard defines a method for requesting vehicle information based on the OBD-II Parameter ID (OBD-II PID). For example, information such as the engine speed (RPM), vehicle speed, and throttle position can be acquired through a predetermined protocol. However, this procedure is limited by the inability to collect information not defined in OBD-II.

Research is thus underway for developing methods for acquiring and using vehicular information not supported by the standard OBD-II. For example, studies have been conducted on acquiring proprietary information by analyzing CAN messages. Some researchers have disassembled vehicle ECUs for packet injection and testing during reverse engineering [24,25]. The transmission of various CAN messages to an ECU enables not only monitoring of the vehicle condition but also its operation. Studies have also been conducted on transmitting messages to ECU in a controlled environment to prevent the vehicle from being driven. However, there is a disadvantage in terms of the time and effort required to generate many CAN messages.

Representative works on the analysis of CAN messages have classified different unknown information and investigated variations in their values [26,27,28]. However, the analysis and interpretation of the acquired sensor information sometimes require expert knowledge. Others have attempted to extract unknown information from data obtained through standard OBD-IIs [29,30]. For example, the wheel rotation speed may be deduced from vehicle speed information obtained from an OBD-II. However, only information with a similar pattern as the OBD-II can be extracted by this means. Moreover, expert knowledge is still required to analyze the information with a similar pattern.

In this paper, we present a novel system for the automatic extraction of vehicle information by analyzing CAN frames collected from the OBD port of the vehicle. Unlike conventional methods that require final analysis by experts, our system has the advantage of enabling automatic extraction of vehicle information when driven without human intervention. The proposed system also contributes to the general technique for analyzing CAN messages applicable to various vehicle manufacturers and models. We also present the development of data collection devices for our system and show the results of collecting data from ten different vehicle types. Our system can operate in semi-automatic and automatic data extraction modes, and experiments are conducted to verify performance in both modes. In particular, the contributions of this study are as follows:

1. We present sensor-based automatic extraction approaches to extract proprietary information, including brake pedal, gear position, odometer, turning signal, and steering wheel.
2. We suggest new techniques for the extraction of in-vehicle information. The techniques can estimate driving status by utilizing the sensor values as input and select the final answer using the scoring algorithm and distance matching.
3. Evaluations using naturalistic driving data are conducted, and the results demonstrate the feasibility of our approaches.

## 2. Overview of the Proposed System

**Figure 1:**
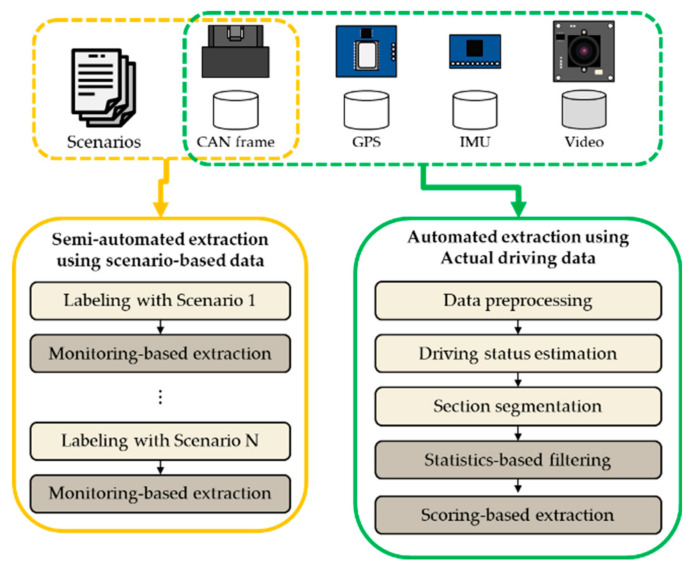
Overview of the proposed in-vehicle information extraction system with two possible data extraction techniques.

As cars became electronic and drivers’ safety concerns increased, all vehicles provide a common way to communicate with the in-vehicle network. We designed a system to extract vehicle information by collecting data from the vehicle network. The proposed system is composed of two parts, with the first part used for the collection of CAN frames, and the second for the extraction of vehicular information. The first part includes a data acquisition system (DAS) that can be installed in the vehicle. From the data collected by the DAS, the second part of the proposed system extracts in-vehicle information. Overall, the system converts the raw CAN frames with unknown meaning into vehicular information that can be analyzed. Figure 1 shows an overview of the system. The upper part illustrates the process of creating a dataset based on the data collected by the DAS. The lower part illustrates the process of extracting information such as the brake pedal position, gear position, steering wheel angle, odometer reading, and turn signals through analysis of the collected raw CAN frames. As indicated in Figure 1, both semi-automatic and automatic data extraction techniques are proposed in our system.

### 2.1. Hardware Design

**Figure 2:**
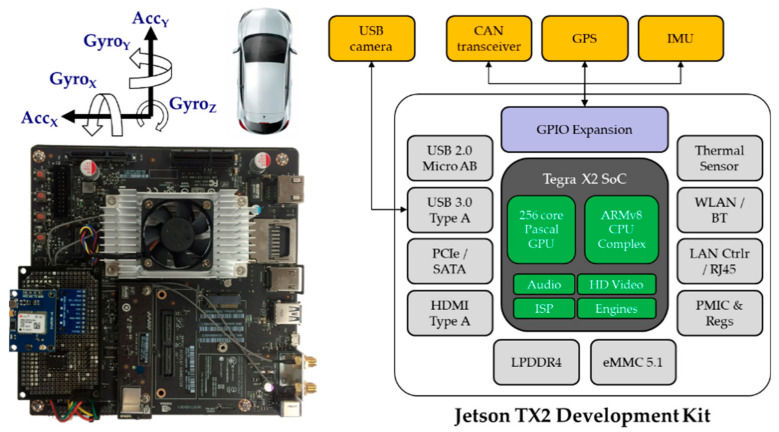
Structure of the DAS of the proposed system.

We designed an embedded DAS hardware, as shown in Figure 2, for installation in vehicles to collect data to implement the proposed system. The DAS design of the proposed system is based on a previous work [31] using Linux-based hardware for collecting vehicle data. The reference system includes a CAN transceiver, GPS, and IMU but has a lower performance than the embedded DAS. The proposed design integrates additional components such as a transceiver for CAN communication, GPS, inertial measurement unit (IMU), and USB camera on a development board using the Nvidia Tegra X2 (Nvidia, Santa Clara, CA, USA) [32] as the core. Table 1 compares the hardware of the two systems. Consequently, the sensing data, such as those acquired by the GPS and IMU are stored alongside the raw CAN data for further analysis. The camera is used for ground truth video recording. Further, the DAS not only stores various data in real-time, but is also capable of extracting CAN information from the data.

### 2.2. Software Design

**Figure 3:**
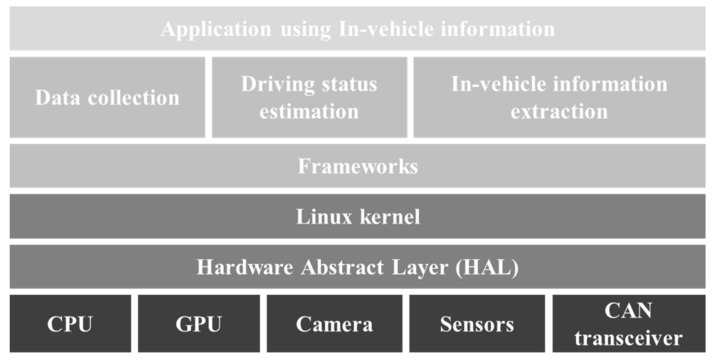
DAS software architecture.

The proposed system consists of the DAS and CAN data extraction software. The data acquisition program was designed to store video data in real-time alongside the CAN data and GPS and IMU sensing data. All the data collected from the vehicle were stored together with timestamps for their synchronization. Both the data acquisition and extraction programs were written in Python. We designed a deep learning inference program for model construction using Keras [33], which utilizes TensorFlow as a backend with the CUDA^®^ Deep Neural Network library. The DAS software is illustrated in Figure 3.

We developed a scenario-based and filter-based method for the semi-automated extraction of vehicle information. According to a predetermined scenario, the specific vehicle information is estimated based on the change in that information during a driving segment. For example, the change in the odometer information after driving 10 km may be used to estimate the odometer information. We developed a method for the gradual reduction of the candidate group size by monitoring data changes produced by the driver’s manipulation of the vehicle.

Further, we estimated the four different driving statuses of vehicles for the automatic extraction of information such as the vehicle acceleration/deceleration, steering wheel angle, brake pedal position, and vehicle travel distance during the data collection. The first three types of information mentioned here were estimated based on the IMU values and GPS speed, while the travel distance was estimated based on the GPS coordinates. The vehicle status information was used as a criterion for dividing the travel segment of the vehicle or for candidate group selection through distance matching (DM).

The CAN frames collected during the automated in-vehicle information extraction process are used to generate candidate groups. The candidates that do not match the statistical characteristics of the target information are then excluded by filtering. If the score calculated by the designed scoring technique does not exceed a certain threshold, an exclusion method is subsequently applied. The scoring technique was designed considering that the classification of data collected from actual vehicles is not as perfect as that of the scenario-based data. In addition, we developed a final answer selection method based on DM to improve extraction performance.

## 3. Semi-Automated Extraction

**Figure 4:**
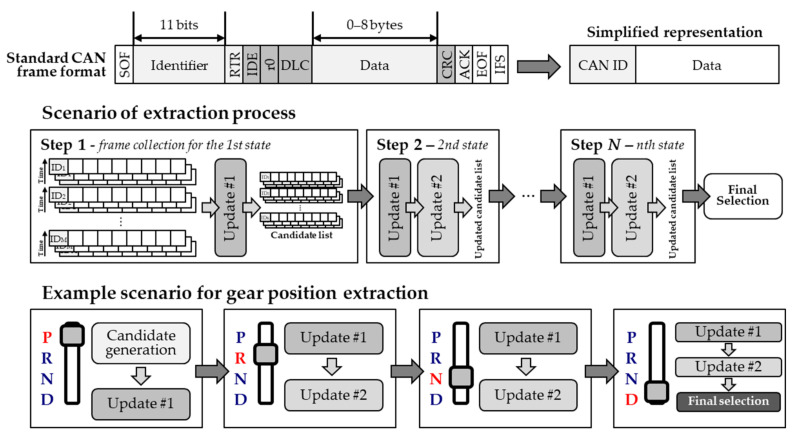
Semi-automated in-vehicle information analysis process.

The semi-automated extraction process is used to obtain vehicle information by limiting the vehicle’s operation to a predetermined scenario. Each scenario is designed based on a strategy that minimizes manipulation, not on the information to be extracted. The driver must operate the vehicle in the prescribed order up to the last step. In the proposed extraction method, a candidate group is set in the first step, and the size of the candidate group is progressively decreased in the subsequent steps. An illustration and examples of the semi-automated extraction process are shown in Figure 4.

### 3.1. Scenarios for Data Collection

In each step defined in the scenario, only CAN frames are collected as input. The last step of the semi-automated extraction process is defined differently depending on the type of information to be extracted. As shown in the example in Figure 4, the last step of the extraction of the gear position is Step 4 because the data are collected in the order P, R, N, and D. Likewise, extraction of the turning signal involves four steps: all off, left on, right on, and all on. The extraction of the brake pedal position involves two steps: on and off. The steps can be progressively increased for the steering wheel and odometer, until extraction is achieved; but, this is possible with only four steps.

For the extraction of any information, the first step always involves the formation of a candidate group. As shown in the example in Figure 4, in the first step of the gear position extraction, the candidates are formed from the frames for P. The size of the candidate group depends on the data length of each CAN frame ID. That is, an ID containing 8 bytes would generate 8 candidates. The data collection time for each step is not fixed but is generally longer than 10 s. After the candidate group formation, it is progressively updated to reduce the number of candidates through continuous monitoring.

### 3.2. Extraction by Data Monitoring

The completion of the extraction process through data monitoring can be divided into two stages. The first update process shown in Figure 4 is premised on the requirement that the data are not changed in this step. For example, when data are collected while the gear position is P, the data that changes during this time will not be related to the gear position. The second update is based on the observed data change in the preceding step. All the candidates with the same collected values for gear positions P and R should be excluded from the candidate group. The final answer is selected by repeatedly performing the two updates described above. Depending on the vehicle type, the number of candidates may not be reduced to one; however, very few candidates remain in most cases and the driver can select the final answer during the test process. The detailed extraction process through data monitoring is presented as Algorithm 1.
**Algorithm 1.** Pseudocode for monitoring-based data extraction*frame*← the CAN frames from collected dataset *n*
← the number of states *candidate*
← the candidate list **for all**
*frame*
**do** *len = frame.length, pre_id = frame.id* *state* = *frame.state* **for**
*i* = 0; *i* < *len*; *i*++ **do**   *can_id* = 10 * *pre_id* + *i*   *val* = *frame.payload*[i]   **if**
*state* = 0 and *can_id*
**not in**
*candidate*
**then**    *temp.id* = *can_id*, *temp.data*[0] = *val*    *candidate.*append(*temp*)   **else then**    *temp* = *candidate*.getById(*can_id*)   **if**
*temp*.*data*[*state*] = NULL **then**    **if**
*val*
**in**
*temp.data*
**then**     *candidate*.remove(*temp*)    **else then**     *temp.data*[*state*] = *val*      *candidate*.replace(*temp*)    **end**   **else if**
*temp*.*data*[*state*] ≠ *val*
**then**     *candidate*.remove(*temp*)   **end**  **end** **end for** **end for**

## 4. Automated Extraction

**Figure 5:**
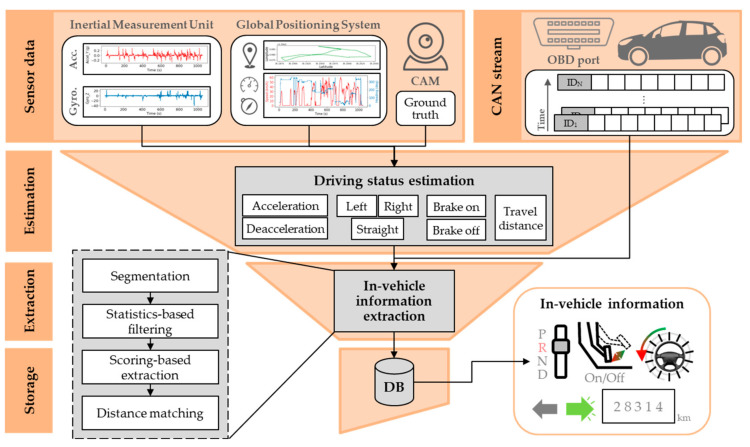
Automated in-vehicle information analysis.

The scenario-based extraction method has the advantage of enabling fast identification of the desired information. However, non-professional drivers may find it difficult to collect the correct data, often inadvertently collecting incorrect data during scenario-based data collection. Therefore, we devised an automated data extraction method based on scoring. The implementation of automated extraction involves five steps. First, the vehicle driving status is estimated. A segment is then separated based on the estimated state. Third, statistical filtering is used to exclude candidates that can be easily eliminated from the candidate group. Next, suitable candidates are selected by a scoring process. Lastly, from among the high-scoring candidates, a DM technique is used to select the final answer. The proposed automatic extraction of vehicle interior information is illustrated in Figure 5. To reduce the complexity of extracting vehicle information in a natural driving environment, we assumed the data collection environment as follows:

1. The driver travels the specified route and always turns on the turn signal before making a left, right, or U-turn.
2. The gear position at the beginning and end of collecting data is at position P.
3. GPS module can always know the location and speed of the vehicle.

### 4.1. Estimation of Driving Status

There are three methods for estimating a vehicle’s driving status, namely, threshold-based method, random forest (RF) method, and long short-term memory (LSTM) method. The threshold-based estimation method is a simple and fast method for using sensor data to recognize specific states exceeding the threshold value. RF is a method for inferring classes based on a multitude of decision trees during system training. RF consists of a combination of randomized decision trees generated based on a random subset of training inputs and random subsets of features. RF selects the final class based on the outputs of the decision tree by learning to determine the test data class. RF votes to find the mode for classification and predicts the average for regression. The LSTM technique was used for classifying the time series data in the deep learning network. The LSTM network consists of several connected LSTM units. A general LSTM unit consists of a memory cell and three gates: an input gate, an output gate, and a forget gate that regulates the flow of information. The input gate controls the flow of new values into the cell, the forget gate controls whether to keep the value, and the output gate uses the cell value for output activation.

**Figure 6:**
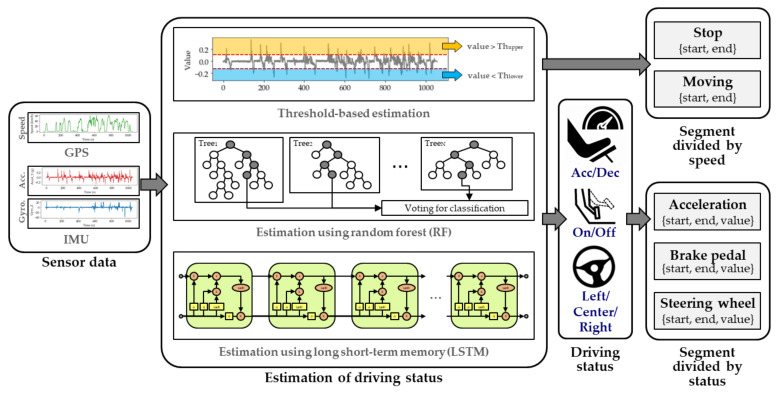
Vehicle driving status estimation and segmentation.

The RF parameters were set through trial and error. The number of trees in the forest was set to 100, maximum depth of the tree was set to 10, and min_samples_split and min_samples_leaf were set as 2 and 1, respectively. The model consists of three consecutive LSTM layers and softmax layers, and the hidden vector size of LSTM is 128. The total number of parameters of the model is about 330,000. Further, the epoch was set to 50, the optimizer was set to adam, and adam’s learning rate was set to 0.001. RF and LSTM enable estimation of the current driving status based on the data inputted during the last 2 s through a sliding window. In the proposed system, the sensor data of the vehicle are not used as learning data but are evaluated through cross-validation. The performance varies with the specific vehicle, being dependent on the height and characteristics of the dashboard on which the data collection device is installed. However, the system was designed for new vehicles using the sensor information of previous vehicles. The process of estimating the driving condition is illustrated on the left side of Figure 6.

### 4.2. Segmentation

In the segmentation process, sensor data are used to divide the CAN frames based on specific criteria. For example, the stop segment consists of a set of statuses in which the vehicle is stopped, while the moving segment consists of a set of statuses in which the vehicle is moving. In the proposed process, five types of segments are created, as shown in Figure 6. The acceleration segment is divided into sections with the same vehicle acceleration status, while the brake pedal segment is divided based on whether the pedal is pressed or not. In addition, the steering wheel segment represents a section in which the direction of the wheel is maintained to the left, middle, or right. Each segment includes a start time, end time, and segment status value, with all the segments having the same estimated status. We set the minimum length of the segments generated by the segmentation process to prevent the presence of noise resulting in the creation of segments that are too small. The section was saved immediately after starting the engine to extract the gear position. We assumed that the gear was in position P when the engine was started.

Each segment was used as an input to the scoring process described in Section 4.4. The acceleration and brake pedal segments were used for the brake pedal extraction process, while the steering wheel segment was used for the turning signal and steering wheel extraction process. The stop segment was used for the odometer extraction and the moving segment for the gear position extraction.

The system calculates the scores of the candidate groups in units of steps made up of segments. If the brake pedal is determined to be pressed, there would be a high probability of the value of the information to be extracted indicating this state. In addition, if the brake pedal is not pressed, it would imply that the vehicle is accelerating, and it can be assumed that the value of the brake pedal is maintained in the corresponding section. In the extraction of other information, the score is similarly calculated using the estimated status in the segment.

### 4.3. Statistics-Based Filtering

Prior to the candidate extraction process, statistics-based filtering is used to eliminate unnecessary computational processes. Among the statistical values of the candidate groups to be filtered, the information of interest is the number of states and minimum amount of change. The number of states is the number of values each candidate has in the collected data and is within 1–255. The minimum amount of change is the minimum value of the difference between the previous value and the current value of each candidate group and is within 0–128. The difference between the two values is not the absolute difference determined by simple subtraction, but the value that must be added or subtracted for a change from the previous value to the current value. For example, when the value of 8-bit data changes from 0xFF to 0x00, the difference is 1, not 255, which is obtained by subtraction. Candidates that do not meet the criteria can be filtered out based on the number of states and calculated minimum amount of change. For example, a candidate with 1 state cannot be a valid candidate because it has a value that never changes. Similarly, all candidates with numbers of states outside the range of the information to be extracted can be excluded. As a result that the brake pedal has only two possible values (on and off), all candidates with numbers of states other than 2 can be deleted. Further, because the turning signal can be off, left, right, or emergency, it can have a minimum of two and a maximum of four states.

The continuity of the data can be ascertained using statistical information referred to as the minimum amount of change. When this amount is 1, the value of a candidate changes in a continuous form. As a result that the information that indicates the angle of the steering wheel has a value that cannot be changed rapidly, it is characterized by continuity. This can be used in the steering wheel extraction process to reduce the candidate group by deleting all candidates with minimum change amounts greater than 2.

### 4.4. Scoring-Based Extraction

Monitoring-based semi-automated extraction can only be used when the current vehicle status is accurately identified. However, the result of determining the driving status based on sensor information may contain errors, and even slight errors in status information can lead to incorrect results. We, therefore, devised a scoring-based extraction method as an error-resistant technique. As mentioned in Section 4.2, we divided the CAN frames into segments for scoring. If the segmentation is properly executed, the correct value for each segment will remain the same. We thus developed a means of scoring how well the selected candidates maintain the same value within a segment. The *i*th candidate $C_{i}={\left\{D_{i,j}\right\}}_{j=1}^{S}$ has data for each step $D_{i,j}={\left\{X_{k}^{\left(i,j\right)}\right\}}_{k=1}^{M_{j}}$, where *S* is the maximum step size of the candidate, and $M_{j}$ is the number of segments included in the *j*th step. The data for each step include data for all the segments within that step. Each segment $X_{k}^{\left(i,j\right)}={\left\{x_{t}^{\left(i,j\right)}\right\}}_{t\;=\;1}^{N_{j,k}}$ contains chronological data between 0 and 255. We defined the number of segments for step *j* as $M_{j}$, the length of the *k*th segment as $N_{k}$, and the total data length of the *j*th step of the *i*th candidate as $W_{j}=\overset{M}{\underset{k=1}{\sum }}N_{k}$. The scoring formula for each step is given by Equation (1), where $m_{i,j}$ is the mode value, which is the most common value in $D_{i,j}$. The mode value is one of the statistically representative values.

(1) $$score\left(D_{i,j}\right)=\;\frac{1}{W_{j}}\overset{M_{j}}{\underset{k=1}{\sum }}\left\{\overset{N_{j,k}}{\underset{t=1}{\sum }}\;\frac{1}{exp\left(\;\left|\overset{\left(i,j\right)}{\underset{t}{x}}-m_{i,j}\right|\;\right)}\;+\sum_{t=2}^{N_{j,k}}\;\frac{1}{exp\left(\;\left|x_{t}^{\left(i,j\right)}-x_{t-1}^{\left(i,j\right)}\right|\;\right)}\;\right\}$$

The score defined by Equation (1) increases with the increasing prevalence of a particular value. The terms on the right-hand side of the equation are designed to have higher values with the repetition of the same value in chronological order. This means that candidates with high scores are similar to the pattern of the driving status to be estimated. In the semi-automated extraction, the driving status is the same, but if the value changes, the candidate is immediately deleted. However, the scoring-based technique eliminates candidates with scores less than the defined threshold $S_{TH}$. We compared the scores for the different steps with $S_{TH}$ and calculated the total score by summing the scores for all steps for candidates with scores guaranteed to be higher than the respective thresholds. The formula for the total score is given by Equation (2). After scoring, the candidate with the highest total score is selected as the final answer.

(2) $$total\_score\left(C_{i}\right)=\overset{S}{\underset{j=0}{\sum }}D_{i,j}$$

### 4.5. Distance Matching for Candidate Selection

As described above, the final answer can be selected and extracted based on the scoring of data into segments. However, we further devised a DM technique to improve extraction performance. When the DM method is applied, the total score-based selection process is replaced with a distance-based scoring. The candidate reduction through scoring for each step is retained in this latter approach. The distance between the graphs are then calculated to select a more suitable candidate. For this purpose, we used the $L^{2}$ norm and dynamic time warping (DTW), which are commonly used graph-to-graph distance calculation methods. First, we applied the $L^{2}$ norm to cases that require consideration of both timing and shape of the graph. The formula for the $L^{2}$ norm is given as follows:

(3) $$L^{2}\;norm\left(A,\;B\right)=A-B_{2}=\sqrt{{\left(a_{1}-b_{1}\right)}^{2}+{\left(a_{2}-b_{2}\right)}^{2}+\cdots +{\left(a_{N}-b_{N}\right)}^{2}}$$

**Figure 7:**
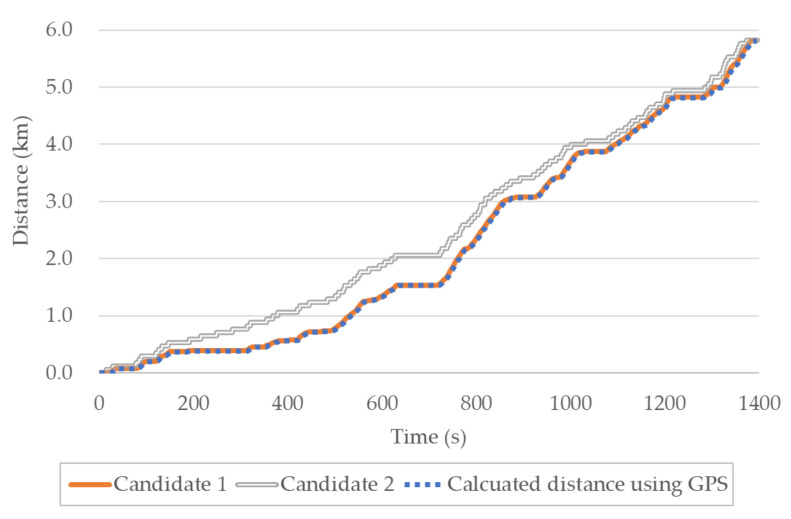
Example of distance matching (DM) for the odometer.

However, it is not easy to obtain the correct result for the odometer based on only the shape of the graph. Hence, FastDTW [34], which has a faster calculation speed, was applied. An example of a graph for the odometer readings are shown in Figure 7. As can be observed, the travel distance of the vehicle calculated from GPS coordinates is more similar to that of candidate 1 than candidate 2. If FastDTW is applied to the two candidates, the value of candidate 1 would be smaller.

We compared the driving state information estimated from the graph of the remaining candidates with that estimated from sensor information using DM. In the example of extracting the brake pedal position, the result obtained from the remaining candidates was compared with that estimated using sensor information. However, a different distance calculation method was used for the extraction of the turning signal. As a result of the lack of a graph for direct comparison of the turning signal, we utilized the fact that the turning signal automatically goes off when the steering wheel is returning to the center position. The time between the turning signal going off, and the steering wheel fully returning to the center position, as estimated from sensor information, was defined as the distance. For comparison of two moments, DM_R was defined as the case in which the moment after the turning signal is turned off is considered; DM_L as the case in which only the previous moment is considered; and DM_B as the case in which both moments are considered. The driver’s manually turning off the turning signal during a lane change or turn may slightly increase the distance value, but it will still be significant.

## 5. Performance Evaluation

### 5.1. Dataset

**Figure 8:**
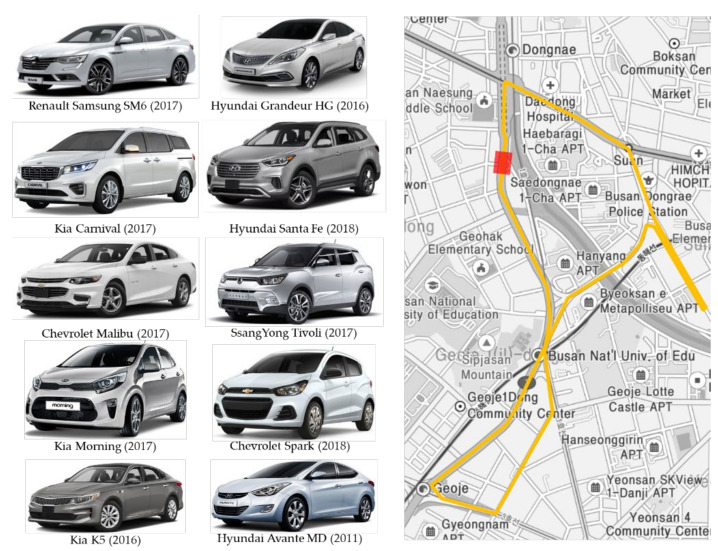
Experimental vehicles and driving route for data acquisition in Busan, Korea.

Data were collected from ten different types of real vehicles to verify the effectiveness of the proposed system. Each vehicle was driven three times over a distance of 5 km in Busan, Korea. The collected data included those for the left turn, right turn, and U-turn. The video camera in the DAS could be used to record various driving experiences in the front of the vehicle for monitoring or to obtain the driving status. The model and year of the vehicles from which the data were collected are shown on the left in Figure 8. The right side of the figure shows the driving route of the vehicles during the data collection. The red square on the map indicates an area where GPS information could not be properly collected.

The details of the data collected during the experiment are summarized in Table 2. Of the 30 driving sessions, obtained by driving each of the ten cars three times over the experimental route, four were excluded due to improper data collection. Only 26 of the sessions were thus considered for the analysis. In addition, the number of raw CAN frames varied among the vehicles. With about 1000–2500 CAN frames collected per second during the total driving time of about 10 h, the overall dataset contained about 59 million CAN frames. The size of the dataset was about 19.46 GB.

### 5.2. Estimation of Driving Status

**Figure 9:**
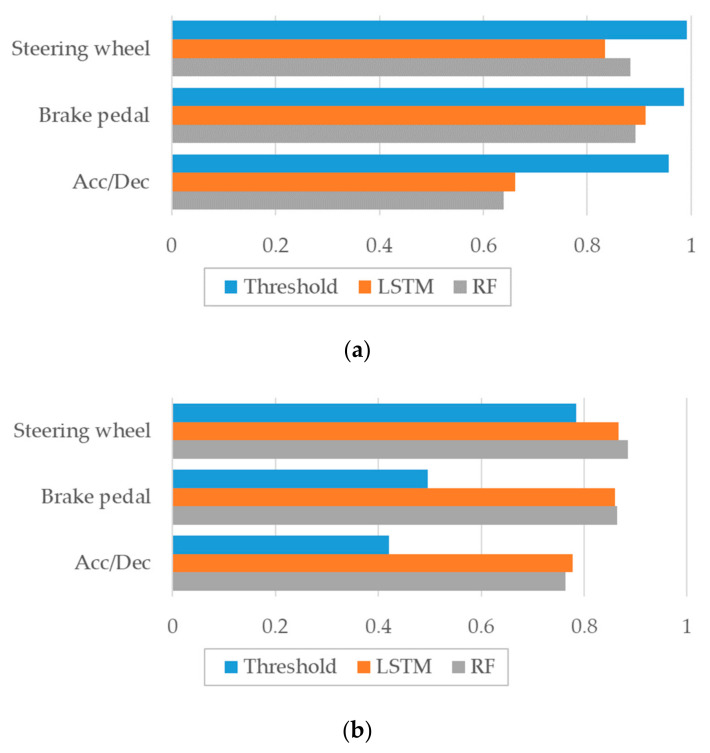
Comparison of the performances of the three proposed estimation methods. (**a**) Precision, (**b**) Accuracy.

We evaluated the performance of the experiment in terms of precision and accuracy. The precision is defined as TP/(TP + FP), and accuracy is defined as TP + TN/(TP + TN + FP + FN), where TP is true positive, TN is true negative, FP is false positive, and FN is false negative. The precision is a value indicating the correct answers among the states estimated as positive. With high precision, the segmentation for positive states becomes more accurate. Accuracy is a metric that considers both positive and negative values, representing the proportion of correct answers among all cases. The performance of estimating the driving status based on sensor data impacts the performance of the vehicle information extraction. Moreover, estimating the driving state of a new vehicle based on sensor data extracted from other vehicles may have performance limitations. We thus evaluated the three proposed estimation methods. Out of the 10 vehicles considered in the experiment, the driving state estimation for one was performed after model training using the nine other vehicles. The performances of the driving condition estimation are shown in Figure 9. Figure 9a shows the estimation precision, while Figure 9b shows the accuracy. As can be observed from the results, threshold-based estimation has high precision but low accuracy, while the precisions of LSTM and RF estimation are a little lower but much more accurate.

Table 3 summarizes the accuracies of the three proposed driving status estimation methods for the different considered vehicles. RF and LSTM-based techniques show more suitable performance than the threshold method. Regarding vehicle acceleration and deceleration, the LSTM-based estimation model performed best with slight differences. However, for the brake pedal and steering wheel conditions, the RF performed slightly better.

### 5.3. Extraction of Information

Before evaluating the automated extraction technique performance, we tested the semi-automated extraction technique to obtain a list of correct results. In cases with erroneous data collection process, the results were incorrect. However, in the iterative test process, the target information was successfully extracted from all the vehicles.

**Figure 10:**
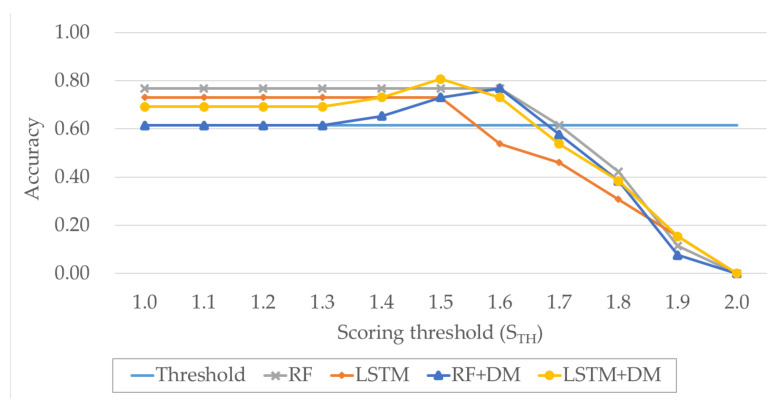
Performance comparison of the different methods for extraction of the brake pedal status.

We defined accuracy as the number of cases in which the correct answer was successfully extracted among all extraction attempts. Furthermore, we defined the ratio of the case with the correct answer among the three candidates with the highest score as Top3 accuracy, and the ratio of the case with the highest score being the correct answer as Top1 accuracy. The performances of the brake pedal status using the automated extraction technique are shown in Figure 10. Different values of $S_{TH}$ (the threshold for scoring) were considered in the experiment. The performance was observed to be lowest when the sensor information was estimated using the threshold value, with the accuracy being 0.62 regardless of $S_{TH}$. When the scoring decided by the segments generated by LSTM and DM was applied, the highest accuracy of 0.81 was obtained. The performances for the other cases were similar, with the accuracy ranging between 0.73 and 0.77. When the $S_{TH}$ value is greater than 1.5, the performance decreases because the braking condition estimation was not perfect, and a more stringent criteria was used to rule out accurate results more often.

Table 4 gives the performances with a correct result among the three candidates with the highest scores (Top3 accuracy), as distinct from when only the final answer was correct (Top1 accuracy). All the methods that were not based on a threshold were found to exhibit high accuracies of up to 0.92. We also observed cases in which the correct result did not have the highest score due to the presence of many other data with patterns similar to that of the brake pedal status. Compared with the Top1 accuracy, the Top3 accuracy was not significantly improved by DM. However, the selection of the final answer through DM contributed to avoiding incorrect results in some Top3 cases.

**Figure 11:**
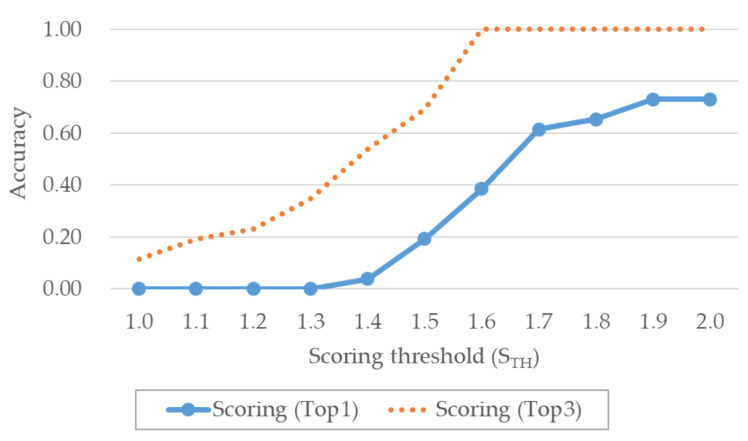
Performance comparison of different automated extraction methods for gear position.

The gear position automated extraction performances are shown in Figure 11. In this case, the extraction success rate was observed to increase with increasing $S_{TH}$. The maximum accuracy for the gear position extraction was 0.73, due to extraction failure in two of the 10 considered vehicles. The moving segment used to extract the gear position is characterized by very high accuracy because it corresponds to when the GPS speed exceeds a certain value. Since the segment used for extracting the gear position has high accuracy, an upward-right graph is drawn. However, because only the gear positions immediately after starting the car and while the car is running are used, the Top1 accuracy is likely to be lower. Nevertheless, evaluation using Top3 accuracy produced correct results for all cases.

**Figure 12:**
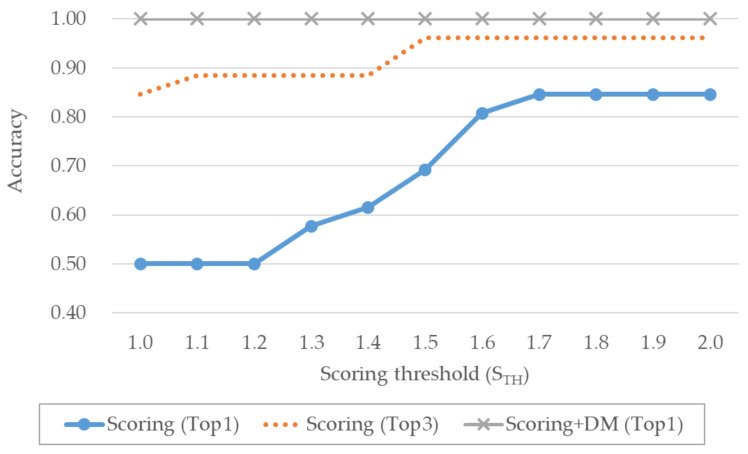
Performance comparison of different automated extraction methods for the odometer.

The odometer automated extraction performances are shown in Figure 12. The odometer extraction utilizes the distance calculated based on the GPS coordinates and Stop segment; therefore, the performance increased with increasing $S_{TH}$. The odometer extraction has high accuracy because the calculated distance value and segment representing the stationary state are used. Therefore, the graph of the extraction performance is made to appear in the upper right. In addition, the performance was greatly improved when DM was applied. When only scoring was applied, the maximum accuracy was 0.85, while the application of DM increased the accuracy to 1.00. The experimental results revealed that the distances calculated using the GPS coordinates and that indicated by the odometer were very similar.

**Figure 13:**
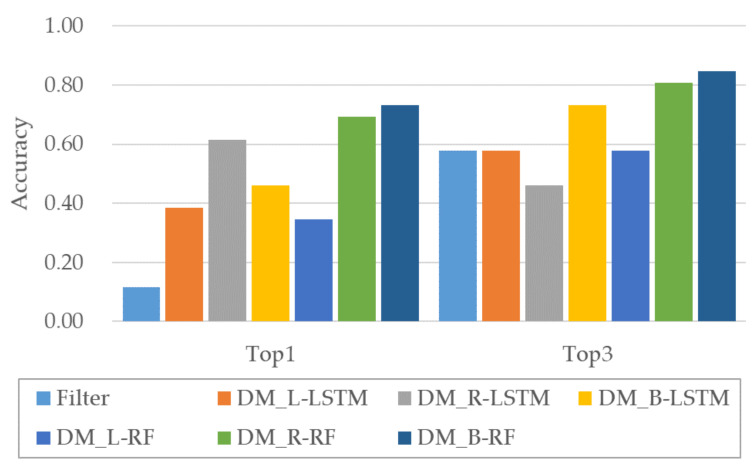
Comparison of extraction performance for turn signal.

The performances of different methods for turning signal extraction were also compared. As shown in Figure 13, the performances were not very good because the turning signal status could not be directly estimated from the sensor information. An accuracy of just 0.12 was achieved by reducing the candidates through filtering using statistical information, implying that the extraction was impossible in most cases. The performance was improved by the application of DM to the filtered results. DM using LSTM and RF produced the highest performances with consideration of the time axis in both directions. Top1 and Top3 accuracies corresponded to the use of RF-based DM considering the time axis in both directions. DM_B-RF produced a Top1 accuracy of 0.73 and Top3 accuracy of 0.85.

**Figure 14:**
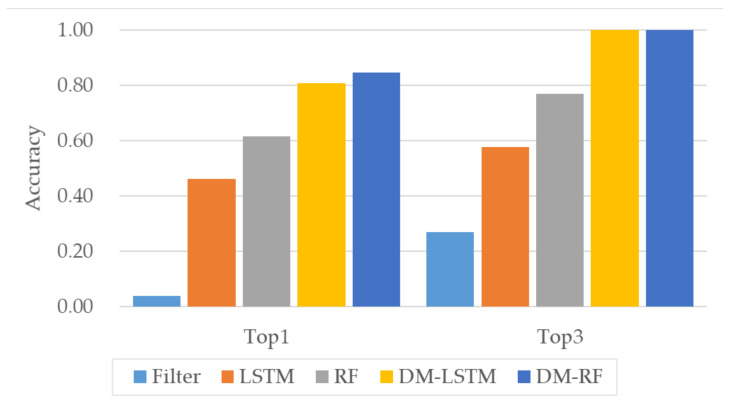
Performance comparison of different methods for steering wheel extraction.

The performances for steering wheel extraction using sensor information acquired from our experiment are shown in Figure 14. As can be observed from the figure, simple statistics-based filtering produced an accuracy of 0.04, indicating extraction failure in most cases. The performance was improved by the application of scoring using sensor information, with LSTM and RF producing accuracies of 0.46 and 0.62, respectively. The best performance was achieved by applying DM without segment-based scoring, with LSTM (DM-LSTM) and RF (DM-RF) producing accuracies of 0.81 and 0.85, respectively. Further, extraction using LSTM- and RF-based DMs produced correct Top3 results for all the considered cases. Overall, the most outstanding steering wheel extraction performance was achieved using RF-based DM.

Table 5 gives the performances for steering wheel extraction without DM. The highest accuracy under this condition was achieved by RF-based scoring (0.62). The performance of each method without DM decreased when $S_{TH}$ exceeded 1.4. The results indicate imperfect estimation of the driving status based on the sensing values, although the proposed system exhibits relatively high extraction accuracy despite the errors in input.

Table 6 gives the results of the automatic extraction of in-vehicle information using the proposed system. In most cases, the correct result was obtained by selecting even only one candidate. Moreover, in each of the three cases with the wrong results, the correct result was observed among the three candidates with the highest scores. The exception was that no turning signal was detected for Tivoli. Data analysis confirmed that the Tivoli turning indicator was an exceptional case in which the state transition occurred twice during the change from on to off. We confirmed the successful extraction of five types of in-vehicle information from the 10 types of vehicles through several experiments.

## 6. Discussion and Conclusions

We developed and implemented a system for analyzing in-vehicle information based on the dataset collected from actual vehicles. In an experimental implementation of the system, we used it to collect CAN frames and sensor data such as GPS and IMU from 10 vehicles. Based on the sensor data, we designed a technique for estimating the driving state of the vehicle and to extract in-vehicle information. Although previous studies have been conducted on the extraction of in-vehicle information, the proposed system unprecedentedly affords automatic extraction. Experimental evaluation of the system performance revealed that, although it was not perfect for all cases, it worked well in most cases. The extraction accuracies for brake pedal, gear position, odometer, turning signal, and steering wheel were 81%, 73%, 100%, 73%, and 85%, respectively and 92%, 100%, 100%, 85%, and 100%, for Top3. The extraction performance for the turning signal was the least, due to the inability to obtain a direct hint from the sensor data. The above-mentioned in-vehicle information extracted by the proposed system are commonly used for vehicle-related research. Thus, this work promises to contribute to the application of existing research to any specific vehicle. We plan to conduct additional research to make the system work on smartphones so that our extraction technology can be practically utilized.

## Figures and Tables

**Table 1:** Hardware specifications of the presented data acquisition system (DAS) and reference system.

Component	Banana Pro (Reference System)	Jetson TX2 Module (Presented DAS)
**CPU**	ARM Cortex-A7 Dual-Core, 1.0 GHz	Denver 2, dual cores, 2.0 GHz Cortex-A57, quad cores, 2.0 GHz
**GPU**	Mali400MP2, 350 MHz	Pascal, 256 cores, 1.12 GHz
**Memory**	DDR3 SDRAM, 1 GB	LPDDR4, 8 GB
**Storage**	MicroSD (TF) card slot	32 GB
**Power**	5 V	5.5–19.6 V
**Size**	92 mm × 60 mm	87 mm × 50 mm

**Table 2:** Details of the dataset collected during the driving experiment.

Component	Sampling Rate	Number of Data	File Size	Description
**Video**	30 frame/s	26	15.56 GB	MP4 format
**CAN**	Variable	54,794,154	2.28 GB	Time, CAN frame (ID + payload)
**IMU**	100 Hz	3,384,297	427.94 MB	Time, 3-axis accelerometer, and 3-axis gyroscope data
**GPS**	1 Hz	33,832	1.62 MB	Time, location, speed over ground, and course over ground

**Table 3:** Accuracies of the three proposed driving status estimation methods.

Vehicle Model	Acceleration/Deceleration			Brake Pedal (on/off)			Steering Wheel (L/C/R)		
	Threshold	LSTM	RF	Threshold	LSTM	RF	Threshold	LSTM	RF
SM6	0.41	0.79	0.77	0.51	0.84	0.86	0.81	0.91	0.90
Grandeur	0.41	0.74	0.75	0.53	0.90	0.90	0.78	0.88	0.90
Santa Fe	0.42	0.77	0.80	0.52	0.89	0.90	0.84	0.93	0.95
Avante	0.46	0.81	0.82	0.49	0.89	0.89	0.71	0.80	0.81
Carnival	0.38	0.86	0.65	0.52	0.81	0.75	0.76	0.87	0.89
Morning	0.41	0.72	0.76	0.48	0.82	0.82	0.79	0.86	0.90
K5	0.31	0.73	0.76	0.58	0.85	0.87	0.73	0.84	0.85
Malibu	0.47	0.77	0.77	0.46	0.85	0.85	0.79	0.86	0.87
Spark	0.49	0.83	0.82	0.37	0.88	0.89	0.82	0.87	0.88
Tivoli	0.46	0.76	0.75	0.52	0.89	0.90	0.80	0.85	0.89
Avg.	0.42	0.78	0.76	0.50	0.86	0.86	0.78	0.87	0.88

**Table 4:** Top3 accuracies for brake pedal status extraction.

Method	DM	Value of Scoring Threshold (*S*_TH_)										
		1.0	1.1	1.2	1.3	1.4	1.5	1.6	1.7	1.8	1.9	2.0
Threshold		0.73	0.81	0.81	0.81	0.81	0.81	0.77	0.81	0.81	0.81	0.81
RF		0.92	0.92	0.92	0.92	0.92	0.92	0.92	0.77	0.58	0.15	0.00
	✓	0.92	0.92	0.92	0.92	0.92	0.92	0.92	0.77	0.58	0.19	0.00
LSTM		0.88	0.92	0.92	0.92	0.92	0.92	0.73	0.65	0.50	0.19	0.00
	✓	0.92	0.92	0.92	0.92	0.92	0.92	0.85	0.65	0.50	0.19	0.00

**Table 5:** Performance of steering wheel extraction without DM.

Method	Metric	Value of Scoring Threshold (*S*_TH_)										
		1.0	1.1	1.2	1.3	1.4	1.5	1.6	1.7	1.8	1.9	2.0
RF	Top1	0.62	0.62	0.62	0.62	0.62	0.58	0.23	0.08	0.00	0.00	0.00
	Top3	0.77	0.77	0.77	0.77	0.77	0.69	0.27	0.08	0.00	0.00	0.00
LSTM	Top1	0.46	0.46	0.46	0.46	0.46	0.42	0.31	0.23	0.08	0.00	0.00
	Top3	0.58	0.58	0.58	0.58	0.58	0.50	0.31	0.23	0.08	0.00	0.00

**Table 6:** Successfulness of each automatic extraction method using sensor information.

Vehicle Manufacturer	Vehicle Model	Turning Signal		Steering Wheel		Brake Pedal		Gear Position		Odometer	
		Top1	Top3	Top1	Top3	Top1	Top3	Top1	Top3	Top1	Top3
Renault	SM6	✓	✓	✓	✓	✓	✓	✓	✓	✓	✓
Hyundai	Grandeur	✓	✓	✓	✓	✓	✓	✓	✓	✓	✓
	Santa Fe	✓	✓	✓	✓	✓	✓	-	✓	✓	✓
	Avante	✓	✓	✓	✓	✓	✓	✓	✓	✓	✓
Kia	Carnival	✓	✓	✓	✓	✓	✓	✓	✓	✓	✓
	Morning	✓	✓	✓	✓	✓	✓	✓	✓	✓	✓
	K5	✓	✓	✓	✓	✓	✓	✓	✓	✓	✓
Chevrolet	Malibu	✓	✓	✓	✓	✓	✓	-	✓	✓	✓
	Spark	✓	✓	✓	✓	-	✓	✓	✓	✓	✓
SsangYong	Tivoli	-	-	✓	✓	✓	✓	✓	✓	✓	✓
